# Successful Treatment in a Child with Anaplastic Large Cell Lymphoma and Coexistence of Pulmonary Tuberculosis

**DOI:** 10.1155/2013/928701

**Published:** 2013-06-13

**Authors:** Margarita Baka, Dimitrios Doganis, Apostolos Pourtsidis, Maria Tsolia, Despina Bouhoutsou, Maria Varvoutsi, Katerina Strantzia, Helen Kosmidis

**Affiliations:** ^1^Oncology Department, “P & A. Kyriakou” Children's Hospital, Thivon & Levadias, 11527 Athens, Greece; ^2^Second Department of Pediatrics, National and Kapodistrian University of Athens School of Medicine, “P & A. Kyriakou” Children's Hospital, Athens, Greece; ^3^Pathology Lab of “P & A. Kyriakou” Children's Hospital, Thivon & Levadias, 11527 Athens, Greece

## Abstract

A 13-year-old girl was admitted to our department with a history of severe pain of her left axilla and fever. On physical examination, a block of lymph nodes in her left axilla, diffuse papular rash, and red-violet swelling of her supraclavicular and subclavian region were noted. Imaging investigations revealed left axillar and supraclavicular lymphadenopathy and a small nodular shade in the upper lobe of her left lung. A biopsy from an axillary lymph node established the diagnosis of anaplastic large cell lymphoma (ALCL), whereas DNA of *Mycobacterium tuberculosis* was detected by polymerase chain reaction (PCR) in the same tissue biopsy. Patient was started on chemotherapy for ALCL and achieved remission of all initially involved fields. Nevertheless, two new nodular lesions were detected in the left lower lobe. Biopsy revealed granulomas, and PCR was positive for *M. tuberculosis*. Our patient received treatment with the combination of isoniazid and rifampin (12 months), pyrazinamide (the first 2 months), and maintenance chemotherapy for her ALCL for one year simultaneously. Four years later, she is disease free for both mycobacterial infection and lymphoma. We are reporting this successful management of mycobacterial infection in a patient with ALCL despite intensive chemotherapy that the patient received at the same time.

## 1. Introduction

Children with cancer have an increased risk for life-threatening infections due to their underlying illness and intensive anticancer treatment as well [1]. Certain types of cancer, particularly Hodgkin's disease and non-Hodgkin's lymphoma, are associated with impaired cellular immunity which may persist even after the end of treatment while the disease is in remission [1, 2]. Chemotherapy in these diseases is an additional factor of altered cellular immunity [1]. Although the majority of infections in children with cancer are caused by bacteria, infections due to mycobacteria are also reported and may be severe and life threatening [1, 3]. In this report we describe a case of pulmonary tuberculosis (TB) in a girl with Anaplastic Large Cell Lymphoma (ALCL).

## 2. Case Report

A 13-year-old girl was admitted to our department with a history of severe pain of her left axilla and limitation of her left arm active movements during the last 3 weeks. The parents reported no history of any other associated symptoms apart from fever of 39°C three days after the first appearance of pain. No history of decreased appetite was reported. No prior surgeries or medical problems were also mentioned. On physical examination, she was well appearing, her vital signs were within normal limits, and she had a grossly normal neurologic examination. Examination of the head and neck was normal. A block of lymph nodes in her left axilla, small cervical lymph nodes, diffuse papular rash, and red-violet swelling of her supraclavicular and subclavian region were seen. Her lungs were clear to auscultation and she moved air well and without effort. She had no concerns in terms of pulmonary issues recently. The remainder of the review of systems in terms of social, neurologic, dermatologic, cardiovascular, gastrointestinal, urologic and musculoskeletal systems, was negative.

Under the hypothesis of an infectious disease, a first course of oral antibiotics was initiated by her pediatrician without any improvement concerning her lymphadenopathy. Therefore, ultrasound and computed tomography investigations took place and revealed left axillar and supraclavicular lymphadenopathy as well as a small nodular shade in the upper lobe of her left lung. At the same time a chest X-ray was normal. In terms of complete blood count, differential, electrolytes, and other biochemical parameters she had a normal set of laboratory tests. However, her LDH, ESR, and uric acid were 561 U/L, 80 mm/hr and 31 mg/L, respectively. Bone marrow aspiration, spinal fluid, bone scanning, and Tuberculin Sensitivity Test (TST) were also performed and were negative.

A biopsy from an axillary lymph node was performed and the diagnosis of ALCL (LCA, MCHL-1, EMA, CD30, and ALK-1 positive) was established. Patient was started on chemotherapy for high risk patients according to current protocol for ALCL (International Protocol for the Treatment of Childhood Anaplastic Lymphoma—ALCL-99). Several days later and after the patient had already started chemotherapy for her lymphoma, DNA of *Mycobacterium Tuberculosis* was detected in the tissue biopsy according to polymerase chain reaction (PCR). In the light of the negative TST, the negative history regarding patient's family, and the histologic features of ALCL, the detection of *M. tuberculosis* DNA was considered as a false-positive PCR test result and our patient continued her chemotherapy courses without administration of anti-TB treatment. 

**Figure 1:**
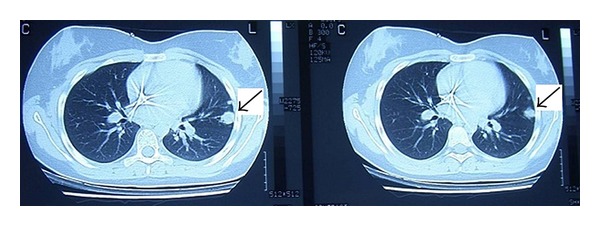
Computed tomography investigation: nodular lesions in the left lower lobe of the lung.

Our patient achieved remission (complete resolution of imaging findings) of all initially involved fields 4.5 months after the diagnosis with (1) an initial course of intravenous dexamethasone and cyclophosphamide and intrathecal infusion of a combination of methotrexate, cytarabine, and cortisone and (2) alternating 3 cycles of AM/BM regimens every 3 weeks (AM: dexamethasone, and high dose of methotreaxate, cytarabine, etoposide, ifosfamide, BM: dexamethasone, cyclophosphamide, doxorubicin, high dose of methotrexate). Nevertheless, two new nodular lesions (with a diameter of 1.5 cm and 0.3 cm, resp.) were detected in the left lower lobe of the lung according to both the X-ray and computed tomography investigations; Figure 1. Biopsy from the lesions revealed granulomas and PCR was positive for *M. tuberculosis*. A TST was repeated and remained negative.

On count of the new biopsy findings she received treatment with combination of isoniazid, rifampin, and pyrazinamide for two months and isoniazid, rifampin for ten months (long-term administration because of immunosuppression). During these twelve months our patient received also maintenance chemotherapy (vinblastine per week) for her ALCL. At the end of treatment both lymphoma and infectious lesions were in remission. Four years from the diagnosis she is disease free for both mycobacterial infection and lymphoma.

## 3. Discussion

We presented a girl with ALCL and coexistence of pulmonary tuberculosis. ALCL is a distinct form of non-Hodgkin lymphoma (NHL) and presents with particular clinical characteristics. There seems to be a predominance of male gender, particularly in cases of positive expression of ALK protein in which the ratio of boys to girls is 3 : 1. Patients may have an indolent phase consisting of a long history of mild lymphadenopathy and fever until finally progression takes place. This type of lymphoma may mimic other diseases and in contrast to adults the disease in children is rarely confined to the skin [4, 5]. 

ALCL is a mature T-cell lymphoma [6] and accounts for 10%–15% of all childhood lymphomas [7]. According to the results of Children's Cancer Group, systemic ALCL is characterized by advanced disease at presentation with a high incidence of nodal involvement, frequent association with B symptoms and frequent extra-nodal involvement including skin, lung, bone, and liver [7].

The expression of the ALK protein which is considered as a distinct type of the disease [6] can be demonstrated in 50%–85% of systemic ALCL but is very rare in cutaneous ALCL [4, 8]. Criteria for the diagnosis of ALCL are the characteristic cells with T or null phenotype as well as the detection of CD30, EMA antigens [9]. The detection of ALK1 antibody (Ki-1 (CD30) positive) is an important tool for the identification of ALCL [10].

Factors associated with unfavorable prognosis are the value of LDH > 800 UI/L, the mediastinal and/or visceral involvement (liver, spleen or lung) as well as skin lesions in areas not correlating with lymph node infiltration. The only unfavourable factor detected in our patient was the lung involvement. According to French Society of Pediatric Oncology the 3-year Overall Survival rate (OS) and Event Free Survival rate (EFS) were 83% and 66%, respectively, whereas in the BFM group the corresponding survival rates were 81% and 69% respectively [10, 11]. 

Our patient was found to have Anaplastic Large Cell Lymphoma ALK-1 positive, but at the same tissue biopsy DNA of *M. tuberculosis* was detected. Based on the negative TST, the negative family history and the relatively low incidence of tuberculosis in the general population in Greece (5.2 cases/100,000 people during 2006 [12]) and especially among native Greek children the report for the presence of *M. tuberculosis* DNA was considered as a false positive result and the patient did not receive treatment for tuberculosis.

Tuberculosis is rarely included in the initial differential diagnosis in developed countries due to the low incidence such as 1.6 cases per 100,000 children in the USA [13]. Studies conducted among adults have shown that the incidence is higher in patients with hematologic disorder (240 cases per 100,000 people) and varies according to country of birth—139 cases per 100,000 persons born in U.S. versus 532 per 100,000 in immigrants. Particulary for patients with Hodgkin's lymphoma an incidence of 231 per 100,000 people is reported [14].

The higher incidence of tuberculosis among patients with hematologic malignancies is attributed mainly to the impaired cellular immunity [15–17]. Impaired cellular immunity is related to the malignancy and the use of corticosteroids and other chemotherapeutic agents as well [2]. Corticosteroids are well known cytokines release inhibitors [18]. In the literature, patients with hairy cell leukemia appear to have an increased risk for infection with atypical mycobacteria [19].

In our patient, *M. tuberculosis* DNA was detected at the same time with the diagnosis of lymphoma and therefore there was no association between the infection and the immunosuppression because of chemotherapy although the patient might have been immunosuppressed because of the disease [2]. Furthermore, upon completion of intensive chemotherapy all the initially involved lesions were in remission, indicating that both the lymphadenopathy and the initial lung lesion were lymphoma infiltrations and not associated with TB disease.

It is remarkable that, despite intensive chemotherapy, the course of infection was slow and not aggressive since only two small pulmonary nodular lesions developed in a different location. Furthermore, all lymph node infiltrations were in remission after chemotherapy, confirming our initial approach that the lymphadenopathy was only manifestation of the lymphoma and suggesting, in retrospect, that *M. tuberculosis* might just be present in the lymph nodes causing subclinical infection and not active disease. 

Additionally, TST was negative before as well as after the intensive phase of chemotherapy. This should be attributed initially to the impairment of cellular immunity from the disease itself and then both to the disease and chemotherapy implications [20]. Further immunosuppression caused by chemotherapy possibly resulted in “activation” of mycobacteria and development of visible lesions during the imaging investigation.

The diagnosis of TB is often delayed among children, because of a low index of suspicion and limitations of commonly available diagnostic tests. Moreover, children are less likely to have positive microbiological findings from sputum examination whereas immunocompromised children are also less likely to have a positive TST [15, 20]. In questionable cases computed tomography may provide more detailed information compared to plain chest radiographs. In our patient, both plain radiographs and computed tomography revealed the pulmonary lesions. Moreover, combined positron emission tomography/CT scan may be useful especially for the diagnosis of extrapulmonary disease [21].

Recently, new immunodiagnostic methods such as the interferon-gamma release assays (QuantiFERON-IGRAs) have been introduced for the diagnosis of *M. tuberculosis* infection. Nevertheless, the use of these methods especially for immunocompromised children remains controversial and they should be used in combination with other methods [22]. Early detection of infection in adult relatives is particularly important apart from the early diagnosis.

The management of mycobacterial infection in children with cancer is a challenge. Nevertheless, there is evidence that treatment is successful both in immunocompetent and in immunocompromised patients [16, 23, 24]. It is well known that children who undergo treatment for malignancies are at higher risk for infection with both typical and opportunistic pathogens [2, 25]. Authors have reported cases of pulmonary tuberculosis in children with lymphoma and more frequently with leukemia [15, 26]. The clinical manifestations of tuberculosis and lymphoma with pulmonary involvement are similar and therefore differential diagnosis is problematic. Tuberculosis should be taken into consideration especially in endemic countries [27]. Extrapulmonary disease is relatively more common in younger patients with tuberculosis and often these cases are misdiagnosed as cancer [28]. On the other hand, authors have reported cases in which the diagnosis of a mycobacterial infection was initially set but a lymphoma was finally detected [29].

The coexistence of lymphoma and mycobacterial infection at the time of diagnosis and particularly in children is rare in the literature. Cases of Hodgkin lymphoma have been mainly reported whereas *M. tuberculosis* or atypical mycobacteria infection as a complication of chemotherapy is more commonly seen [16, 26, 27, 30]. Karakas et al. have reported 14 patients with Hodgkin lymphoma in whom tuberculosis preceded the diagnosis of malignancy in 3 of them, the diagnosis was set at the same time in 2; in 7 of them the infection was diagnosed while on chemotherapy and in 2 after the cessation of lymphoma treatment [27]. Nevertheless, an adult case has been reported with coexistence of ALCL (Ki-1 positive expression) and tuberculous pyothorax [31].

An extensive review has been recently published regarding the coexistence of tuberculosis and cancer concerning mainly adult patients. The researchers conclude that the coexistence is random in the majority of cases in the context that both diseases are common. Nevertheless, extensive reference has been held concerning the possible “carcinogen” role of *M. tuberculosis* which is related to the DNA damage that the inflammatory changes may cause. The authors suggest that further research in this field is needed. Finally they suggest evaluation for mycobacterial infection before the start of chemotherapy even a positive for cancer biopsy is available since starting chemotherapy without taking into account the possible TB infection may lead to activation of a potentially lethal infection [32]. 

The American Academy of Pediatrics recommends that TST should be performed prior to the initiation of immunosuppressive therapy in all children and prophylactic isoniazid treatment given to those with a positive response once active disease has been excluded [33]. Children who received more intensive chemotherapy may require prolonged treatment for tuberculosis compared with children receiving maintenance therapy.

Our patient eventually received treatment for active TB as well as maintenance chemotherapy for one year and she is in remission of the lymphoma and free of TB disease. This case demonstrates the need for a full and precise medical and family history as well as an exhaustive investigation including TST, full imaging, microbiological, and histological examination of suspect lesions before starting chemotherapy. It is obvious that children with cancer are a high risk group who would benefit from screening for tuberculosis.
